# COVID-19 and older adults in Africa: Social workers’ utilization of mass media in enforcing policy change

**DOI:** 10.1177/0020872820941748

**Published:** 2020-09

**Authors:** Tracy BE Omorogiuwa

**Affiliations:** University of Benin, Nigeria

**Keywords:** Community-based, COVID-19, mass media, older adults, policy change, social work

## Abstract

Dominating headlines in the last few months, global attention has been fixed on the coronavirus pandemic given its rampaging impact on social events and human affairs. More than any other groups, older adults have been disproportionately affected by the deadly contagion. This situation poses legitimate concerns to the social work profession, whose mandate is to liberate vulnerable people and promote social development. Although the COVID-19 pandemic has continued to take a devastating toll on older adults in the short-term, its long-term consequences may be far more profound unless urgent attention is directed to mitigate this situation. Given the promulgation of social distancing and shutdowns among a number of African countries, many social workers have found it increasingly difficult to address the difficulties faced by older adults. This article canvasses for the utilization of the mass media in initiating policy response to the challenges of older adults throughout the continent.

## Introduction

Commanding features over the most recent couple of months, worldwide consideration has been fastened on the coronavirus pandemic, given its rampaging sway on social events or get-togethers and human issues. More than some other gatherings, more seasoned grown-ups/elderly persons have been excessively influenced by the destructive infection. This circumstance presents authentic worries to the social work calling, whose order is to free powerless individuals and advance social turn of events. In spite of the fact that the COVID-19 pandemic has kept on negatively affecting older adults, its long term impacts might be far significant except if critical consideration is taken. Although studies abound as to the importance of the mass media in initiating policy change (De Vreese, 2005; Eveland, 2002; Iyengar, 1991; Jerit et al., 2006; Lawrence, 2000; Lee et al., 2008; Schudson, 2003; Walgrave and Van Aelst, 2006), there is slim evidence in Africa pointing towards social workers’ usage of the media in policy advocacy for vulnerable groups (Amadasun, 2020a; International Federation of Social Workers [IFSW], 2020). Before considering how social workers can ‘activate’ this powerful but often overlooked tool, we examine the grievous effects of the pandemic on older adults in Africa.

## How COVID-19 affects older adults in Africa

Studies have shown that many older adults throughout the continent are dependent on their children and relatives, as well as reliant on the informal economy (such as the agriculture and fishing industries, including engaging in petty trade and menial jobs) for survival (Omorogiuwa, 2017). Customarily, families are the bedrock of caregiving to older adults, but recent development, wrought by the forces of globalization, has collapsed Africa’s age-long kinship system, thereby elevating the vulnerability of this at-risk group all the more (Omorogiuwa, 2020). Promulgations of social distancing and mandatory self-isolation (in a bid to curtail the spread of the contagion) have resulted in a negative trade-off, affecting the livelihood of older adults. This has been exacerbated in light of emerging reports attesting to inadequacy in palliative measures (Human Rights Watch, 2020; Okojie, 2020), including the diversion of these limited resources by affluent officials (Daily Trust, 2020; Hassan, 2020). Suffice to assert that this situation is trenchant owing to the non-inclusion of social workers in the administration of welfare packages. Furthermore, as the global economy plummets, exceeding the Great Recession of 2007–2009 (Gopinath, 2020; International Monetary Fund, 2020), mass layoffs, including cuts in paychecks and unemployment, have ensued.

Implicit in the foregoing repertoire is that many families that hitherto had maintained the traditional values of kinship care may be forced to sever ties with their aged relatives and parents. Equally, on perceiving the dire situation and the challenges their caregivers face, older adults may feel compelled to ‘relieve’ their relatives of caregiving duties. Again, the largely dilapidated state of the public healthcare infrastructure suggests that many senior citizens will be unable to secure healthcare in emergency situations. This is aggravated by reports of grossly inadequate test kits, ventilators, personal protective equipment, and isolation centres – all essentials for treatment and recovery (Finnan, 2020; Médecins Sans Frontières, 2020). Disturbingly, the resultant effect of this situation is that many older adults may have contracted the virus but are unaware of such reality. This, on the whole, may spell doom for the general population. This means that, as core stakeholders, social workers have got more reasons to be worried (rightly so, since we are most affected by the pandemic) (Amadasun, 2020b) as we cannot afford to lose more of our highly resourced but undervalued citizens than we already have done.

## Effecting policy change through the mass media

On a positive note, the COVID-19 pandemic has brought to the fore, more vociferously than ever, the imperative of urgent policy response to the challenges of senior citizens in Africa. Pointedly, these challenges, as hinted earlier, range from lack of (in some cases) and inadequate (in many cases) social protection for older adults, to insufficient geriatric healthcare institutions and community-based care. These facilities are needed in abundance throughout the continent, and they require the services of multi-professionals, including social workers. In fact, given the biopsychosocial focus of the social work profession, many practitioners would be instrumental in this regard (Amadasun and Omorogiuwa, 2020). It is against this background that social workers must be at the vanguard of promoting policy change in the context of the challenges faced by older adults in Africa, through the instrumentality of the mass media.

## Operationalizing our professional action

**Figure 1.**
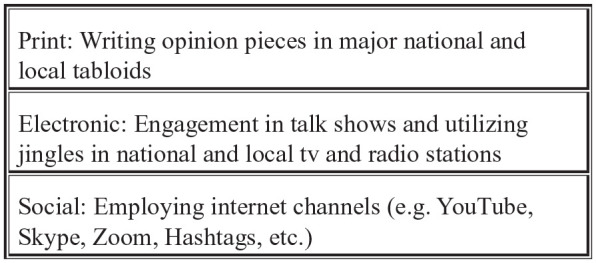
Tripartite media tool.

Given the restrictions on social gathering, social workers can drive their actions through the tripartite (Figure 1) layer of the mass media.

The overall aim of our actions should be premised on education, counselling and advocacy. Via education, we can inform the public and policymakers about the plight of older adults, which is aggravated by the indiscriminate allocation of palliative measures, as well as their deprivation of access to medical care. Through this role, social workers can restate their expertise in social welfare administration and reclaim their position in this regard. Through the counselling role (Abiodun et al., 2011; Omorogiuwa, 2016), we can consolidate the resilience displayed so far by older adults by emphasizing their strengths, while urging policymakers to step up action for service delivery. In the context of advocacy, social workers have an integral role to play and immense responsibility to assume. Researchers have identified three policy fronts necessary for making an impact: ad hoc, intermediate and long-term (Amadasun, 2020b; Amadasun and Omorogiuwa, 2020; Finnan, 2020; IFSW, 2020; Omorogiuwa, 2016). In specific terms, Amadasun (2020b) defined ad hoc policy action as designed to address the immediate needs of older adults, facilitated through cash transfers or in-kind services (e.g. food deliveries). Intermediate policy response is aimed at evaluative action and corrective purpose (Amadasun and Omorogiuwa, 2020). According to these researchers, social workers can set out to evaluate the effectiveness of ad hoc policy intervention with a view to consolidating achieved gains and/or to making corrections in the event of shortfall in policy objectives. Long-term policy response is construed as actions aimed at eliminating structural impediments (Amadasun, 2020b). In this sense, social workers should advocate for alternative means of care (e.g. community-based approach to care, not as a replacement but as a complement to existing institutional care) in order to decongest the limited and overstretched public healthcare facilities, while canvassing for investments in socioeconomic and public health infrastructures.

## Conclusion

The coronavirus pandemic has restated the necessity of urgent policy response to older adults in Africa. Although the impact of the mass media in initiating policy change at both micro and macro levels is well noted, social workers, as agents of social change, have scantly deployed this channel to working with older adults in Africa. This article has underscored the imperative of employing the mass media in (1) supporting older adults, (2) raising awareness about their challenges and (3) engaging in policy change through investments in social protection programmes and alternative means of care to older adults. Taken together, it is believed that social workers can play a pivotal role in improving the social conditions of older adults in Africa, not only during the pandemic but also in its aftermath.

Furthermore, this study has significant implications for social workers in the international arena and in healthcare settings. Using the mass media, social workers can help empower the older adults not only in Africa but also in other parts of the world, by striving to understand cultural diversity, appreciate cross-cultural knowledge and be open to indigenous ways to problem-solving. Healthcare social workers should be sensitive to alternative models whose focal point is built around restoration through strengths and relationship promotion instead of depending solely on the overly formalized clinical outlook and its concomitant pathological and disempowering language. Drawing on service-users’ strengths by promoting collaborations, story-telling and knowledge sharing, especially in a group context, is one way practitioners worldwide can empower and help older adults recover from and/or cope with difficult times as typified by the current pandemic.
